# Outcome of repair of obstetric anal sphincter injuries after three years

**DOI:** 10.1016/j.ijgo.2014.04.013

**Published:** 2014-10

**Authors:** Annette J. Reid, Andrew D. Beggs, Abdul H. Sultan, Anne-Marie Roos, Ranee Thakar

**Affiliations:** a Department of Obstetrics and Gynecology, Croydon University Hospital, Croydon, UK; b Colorectal Research Fellow, Croydon University Hospital, Croydon, UK

**Keywords:** Bowel symptoms, Childbirth, Endoanal scan, Fecal incontinence, Obstetric anal sphincter injuries, Third-degree tears, Vaginal delivery

## Abstract

**Objective:**

To prospectively assess change in bowel symptoms and quality of life (QoL) approximately 3 years after primary repair of obstetric anal sphincter injuries (OASIS).

**Methods:**

Between July 2002 and December 2007 women who attended the perineal clinic at Croydon University Hospital, UK, 9 weeks following primary repair of OASIS were asked to complete the Manchester Health Questionnaire and a questionnaire to obtain a St Mark incontinence score. All women had endoanal scans at this visit. In June 2008 all women were asked to complete the questionnaires again.

**Results:**

Of 344 patients who responded to the questionnaires and were included in the analysis, long-term symptoms of fecal urgency, flatus incontinence, and fecal incontinence occurred in 62 (18.0%), 52 (15.1%), and 36 (10.5%), respectively. Overall, there was a significant improvement in fecal urgency (*P* < 0.001) and flatus incontinence (*P* < 0.001) from 9 weeks to 3 years. Of 31 women with fecal incontinence symptoms at early follow-up, 28 were asymptomatic at 3 years. However, 33 women developed de novo symptoms. The only predictors of fecal incontinence at 3 years were fecal urgency at 9 weeks (OR 4.65; 95% CI, 1.38–15.70) and a higher St Mark score (OR 1.40; 95% CI, 1.09–1.80).

**Conclusion:**

Following primary repair of OASIS, the majority of symptoms and QoL significantly improve, unless there is a persistent anal sphincter defect. This highlights the importance of adequate repair.

## 1 Introduction

Anal incontinence can have a major impact on a woman’s social, physical, and psychological well-being. The reported prevalence of anal incontinence following obstetric anal sphincter injuries (OASIS) varies, ranging between 15% and 61% [1], [2], [3]. Nordenstam et al. [4] assessed the natural progression of bowel symptoms after childbirth and found that the prevalence of symptoms of anal incontinence significantly increased with time, particularly in women with anal sphincter defects.

Using the internationally recommended classification of OASIS [5], [6], [7], [8], which was originally described by Sultan in 1999 [5], it has been shown that symptoms of anal incontinence become increasingly prevalent on short-term follow-up as the grade of OASIS increases [9]. Furthermore, De Leeuw et al. [3] showed that OASIS grade is an independent risk factor for fecal incontinence in the long-term, although they used a different classification of OASIS. We are not aware of a long-term follow-up study using the current internationally recommended classification [5], [6], [7], [8]. Both at short- and long-term follow-up, an association between internal anal sphincter (IAS) defects and severe symptoms of anal incontinence has been demonstrated [2], [10]. Additionally, combined defects of the IAS and external anal sphincter (EAS) are associated with an increased risk of fecal incontinence [9], [11].

The aim of the present study was to prospectively establish the presence of symptoms, changes in quality of life (QoL), and risk factors for symptoms 3 years after primary repair of OASIS.

## 2 Materials and methods

The present prospective study was carried out in a perineal clinic at Croydon University Hospital, Croydon, UK. Consecutive women who had undergone primary repair of an anal sphincter tear sustained during childbirth and returned to the perineal clinic for follow-up approximately 9 weeks postpartum between July 1, 2002, and December 31, 2007, were included. The study was granted exemption by the London-Surrey Borders ethics research committee. Informed consent was obtained from all patients.

The detailed protocol for the surgical technique used to repair anal sphincter tears has been previously described [1]. Partial thickness external sphincter tears were repaired using an end-to-end technique. Full thickness external sphincter tears were repaired by either end-to-end or overlap techniques based on operator choice and expertise. Internal sphincter tears were repaired using an end-to-end technique.

Patients completed the Manchester Health Questionnaire (MHQ) at their 9-week follow-up visit [12]. From 2004, a St Mark incontinence score was also obtained from information gathered using the St Mark questionnaire [13]. All women had an endoanal ultrasound at this visit.

In June 2008 all women who had sustained OASIS at least 6 months previously were sent the MHQ and St Mark questionnaire again and asked to complete them. In addition, information regarding subsequent deliveries and medical illnesses/operations was collected. Patients who did not respond initially were sent a second questionnaire 2–3 months later, and those who did not respond to this second mailing were contacted by telephone.

The MHQ consists of questions about bowel symptoms, scored from 1 (never) to 5 (all the time), as well as validated QoL questions divided into domains of general health, incontinence impact, role, physical function, social function, personal function, emotional problems, sleep/energy, and severity measures, scored from 0 (never affected) to 100 (always affected). MHQ bowel symptoms were dichotomized. Fecal urgency was considered present when a strong desire to move bowels was present sometimes (score of 3) to all the time (score of 5). Flatus incontinence was present when difficulty controlling wind was present sometimes (3) to all the time (5). Because any frequency of fecal incontinence is considered to be distressing, liquid and solid stool incontinence were considered to be present when leakage of liquid or solid stool had ever occurred. Anal incontinence included one or more of the symptoms of flatus, or liquid or solid stool incontinence. Fecal incontinence was defined as liquid and/or solid stool incontinence.

The St Mark questionnaire [13] scores symptoms of flatus and solid and liquid stool incontinence from 0 (never) to 4 (always). Effects on lifestyle, need to wear a pad, use of constipating medicines, and ability to defer defecation are also assessed, with a total score of 0 indicating perfect continence and 24 indicating total incontinence [13].

Before 2005 endoanal ultrasound was performed using the Leopard system (B&K Medical, Gentofte, Denmark) with the endoanal 10-MHz rotating transducer (Type-1850). From 2005 the Viking 2400 system (B&K Medical) was used with the 13-MHz Type-2050 endoanal probe. Images were reviewed at four levels: puborectalis was reviewed as the first level, and the EAS and IAS were subdivided into deep (proximal), superficial (mid), and subcutaneous (distal) levels [14]. An IAS or EAS defect was defined as any defect of more than 30 degrees present in two of the three levels of the anal sphincter [15].

All data were entered into SPSS version 16 (SPSS Inc, Chicago, IL, USA) for statistical analysis. Pearson χ^2^ and Fischer exact tests were used for comparison of categorical data, and the Mann-Whitney *U* and *t* tests were used for continuous variables. The Wilcoxon signed rank test was used to analyze QoL scores over time. *P* < 0.05 was considered statistically significant. Univariate and multivariate logistic regressions were used to analyze predictors of long-term outcome.

## 3 Results

Of the 539 women who were sent questionnaires in June 2008, 349 (64.7%) responded. A total of 201 (57.6%) completed the questionnaire by post and 148 (42.4%) via telephone. Five respondents were excluded because they had undergone secondary sphincter repair following the index delivery. Therefore, 344 women were included in the analysis. There were no significant differences between respondents and nonrespondents at the first follow-up visit after a mean of 9 ± 6 weeks (Table 1).

**Table 1:** Demographics of respondents and non-respondents at 3-year follow-up.^a^

Characteristic	Respondents (n = 344)	Nonrespondents (n = 192)	*P* value
Parity at index delivery	1.32 ± 0.56	1.24 ± 0.56	0.127^b^
Age at index delivery, y	30.4 ± 4.9	29.5 ± 6.2	0.064^b^
Mode of delivery c			
Normal vaginal delivery	245 (71.2)	128 (66.7)	0.297^d^
Instrumental	99 (28.8)	63 (32.8)	
Grade of tear			
Minor (3a/3b)	270 (78.4)	152 (79.2)	0.586^d^
Major (3c/4)	54 (15.7)	35 (18.2)	
Non-categorized third degree	19 (5.5)	5 (2.6)	
Any internal anal sphincter defect	43 (12.5)	23 (12.0)	0.837^d^
Any external anal sphincter defect	19 (5.5)	13 (6.8)	0.738^d^
Defecatory symptoms (Manchester Health Questionnaire scores) at 9 weeks			
Fecal urgency	2.15 ± 0.96	2.09 ± 0.96	0.403^e^
Flatus incontinence	1.79 ± 0.98	1.69 ± 1.02	0.089^e^
Liquid stool incontinence	1.09 ± 0.38	1.09 ± 0.45	0.396^e^
Solid stool incontinence	1.07 ± 0.36	1.10 ± 0.51	0.807^e^

a Values are given as mean ± SD or number (percentage).

b *t* test.

c Data missing for one nonrespondent.

d χ2 test.

e Mann-Whitney *U* test.

The index delivery (during which the first OASIS occurred) was spontaneous in 239 (69.5%) women; 15 (4.4%) women delivered by forceps, 57 (16.6%) by ventouse, 26 (7.6%) by failed ventouse and successful forceps, and 1 (0.3%) by cesarean following failed instruments. At the index delivery, 138 (40.1%) women sustained a grade 3a tear (< 50% EAS torn), 132 (38.4%) a grade 3b tear (> 50% EAS torn), 24 (7.0%) a grade 3c tear (combined EAS and IAS tear), and 30 (8.7%) a grade 4 tear (tear in anal sphincter complex and anal epithelium). In 19 (5.5%) women, the grade of tear was not specified by the surgeon repairing the tear.

After a mean follow-up of 3.2 ± 1.6 years, 241 (70.1%) women had had no further pregnancies. Of the 93 women who had had a subsequent delivery, 68 (73.1%) had a vaginal delivery and 24 (25.8%) a cesarean delivery (data was missing on one). Of those with subsequent vaginal delivery, information regarding subsequent perineal trauma was available in 59 women. Three (4.4%) women with a subsequent vaginal delivery sustained a further OASIS.

At long-term follow-up, 76 (22.1%) women had anal incontinence of whom 36 (10.5%) had fecal incontinence. Fecal urgency, flatus incontinence, liquid stool incontinence, and solid stool incontinence occurred in 62 (18.0%), 52 (15.1%), 35 (10.2%), and 9 (2.6%) women, respectively, with some having multiple symptoms.

There was a significant improvement in presence of fecal urgency from 9 weeks to 3 years (*P* < 0.001) (Table 2). The finding was similar for flatus incontinence (Table 3).

**Table 2:** Comparison of fecal urgency between follow-up at 9 weeks and after 3 years.^a^, ^b^

Urgency at 9 weeks	Urgency at 3 years		Total
	No	Yes	
No	196 (59.6)	27 (8.2)^c^	223 (67.8)
Yes	73 (22.2)	33 (10.0)^d^	106 (32.2)
Total	269 (81.8)	60 (18.2)	329 (100.0)

a Values are given as number (percentage).

b Pearson χ^2^ = 17.439. *P* < 0.001.

c De novo symptoms.

d Persistent symptoms.

**Table 3:** Comparison of flatus incontinence symptoms between follow-up at 9 weeks and after 3 years.^a^, ^b^

Flatus incontinence at 9 weeks	Flatus incontinence at 3 years		Total
	No	Yes	
No	237 (72.0)	26 (7.9)	263 (79.9)
Yes	43 (13.1)	23 (7.0)	66 (20.1)
Total	280 (85.1)	49 (14.9)	329 (100.0)

a Values are given as number (percentage).

b Pearson χ^2^ = 25.937. *P* < 0.001.

Of 331 women for whom data were available for fecal incontinence, 36 (10.9%) were symptomatic at long-term follow-up; 33 (10.0%) had de novo symptoms. Of the 31 women with fecal incontinence symptoms at early follow-up, 28 were asymptomatic at 3 years. Overall, the change in presence of fecal incontinence symptoms was not significant (*P* = 0.822) (Table 4).

**Table 4:** Comparison of fecal incontinence (liquid and/or solid) between follow-up at 9 weeks and after 3 years.^a^, ^b^

Fecal incontinence at 9 weeks	Fecal incontinence at 3 years		Total
	No	Yes	
No	267 (80.7)	33 (10.0)	300 (90.6)
Yes	28 (8.5)	3 (0.9)	31 (9.4)
Total	295 (89.1)	36 (10.9)	331 (100.0)

a Values are given as number (percentage).

b Two-sided Fisher exact test *P* > 0.99. One-sided Fisher exact test *P* = 0.557.

The grade of tear was analyzed in two groups. Minor tears (n = 270) were of grades 3a and 3b, and major tears (n = 54) of grades 3c and 4. Compared to 9 weeks, there was a significant improvement in urgency and flatus incontinence for both minor and major tears at 3 years (*P* < 0.01). However, there was no significant change in presence of fecal incontinence in women sustaining minor and major tears.

Between 9 weeks and 3 years, individuals without any persistent sphincter defect on endoanal scan (n = 280) showed significant improvement in urgency and flatus incontinence symptoms (*P* < 0.001), but not in fecal incontinence (*P* = 0.714). There was no significant change in all symptoms in women with persistent anal sphincter defects (n = 52). Although 38 (73.1%) of these women remained asymptomatic for fecal incontinence, 7 (13.5%) had symptoms at 9 weeks and another 7 (13.5%) had symptoms at 3 years. These findings were similar for urgency and flatus incontinence (data not shown).

Overall, all women showed a significant improvement (*P* < 0.001) in all domains of QoL at long-term follow-up, apart from in the domains of personal relationships (*P* = 0.536) and sleep (*P* = 0.429). Subgroup analysis of women without anal sphincter defects also showed a significant improvement (*P* < 0.001). However, women with an anal sphincter defect and those who developed de novo fecal incontinence did not show any significant improvement in most QoL domains.

More women who had a subsequent delivery by cesarean had fecal incontinence at long-term follow-up than did those who had a subsequent vaginal delivery (Table 5). Of the five women with fecal incontinence symptoms at 3 years who had had a subsequent cesarean delivery, one had persistent fecal incontinence (at 9 weeks and 3 years) and four had de novo symptoms.

**Table 5:** Comparison of mode of subsequent delivery and fecal incontinence symptoms at 3 years.^a^, ^b^

Mode of subsequent delivery	Fecal incontinence at 3 years		Total
	No	Yes	
Vaginal delivery	66 (71.7)	2 (2.2)	68 (73.9)
Cesarean delivery	19 (20.7)	5 (5.4)	24 (26.1)
Total	85 (92.4)	7 (7.6)	92 (100.0)

a Values are given as number (percentage).

b One-sided and two-sided Fisher exact tests *P* = 0.012.

Risk factors for fecal urgency, flatus incontinence, and fecal incontinence at long-term follow-up were analyzed using univariate and multivariate logistic regression with an inclusion threshold of *P* = 0.05. For both fecal urgency and flatus incontinence at long-term follow-up, the only independent predictors were fecal urgency (odds ratio [OR] 3.19; 95% confidence interval [CI], 1.41–7.22) and flatus incontinence at 9 weeks (OR 3.41; 95% CI, 1.34–8.66) in both univariate and multivariate models.

Factors that increased the likelihood of fecal incontinence at 3 years were a higher St Mark score at 9 weeks (OR 1.40; 95%CI, 1.09–1.80) and the presence of fecal urgency at 9 weeks (OR 4.65; 95% CI, 1.38–15.70). Flatus incontinence at 9 weeks was associated with a reduced likelihood of fecal incontinence (OR 0.11; 95% CI, 0.14–0.93).

## 4 Discussion

In this large, prospective study, the prevalence of fecal urgency and flatus symptoms improved significantly approximately 3 years after primary repair of OASIS. At long-term follow-up, more than one-fifth of women complained of anal incontinence and approximately one-tenth had fecal incontinence. Other studies have reported varying frequencies of anal incontinence, from 15% after 13 years of follow-up [16] to 42% after 2–4 years of follow-up [17]. This variance could be attributed to the use of different questionnaires and outcome measures.

In the absence of a grade A recommended questionnaire [7] to evaluate bowel symptoms at the time the present study was performed, the MHQ (Grade B) was used, which has been validated for QoL [12]. The validated St Mark scoring system was used as an objective assessment of bowel symptoms and was applied during regression analysis to determine predictors of long-term symptoms. Because the St Mark score has been shown to correlate well with subjective assessment of impact on QoL following OASIS [18], and because increasing scores positively correlate with long-term symptoms, it appears to be a good tool to use during postpartum surveillance.

Although the majority of women who had symptoms of fecal incontinence at early follow-up improved, and the majority who were asymptomatic remained so at long-term follow up, the change in the presence of this symptom was not significant. This was probably because the majority of those with fecal incontinence at long-term follow-up complained of de novo symptoms. An explanation for these new symptoms could be ongoing fecal urgency that may result in leakage if women are unable to defer a bowel action—a hypothesis supported by the finding in the present study that fecal urgency at 9 weeks was a predictor of fecal incontinence in the long term.

Subsequent delivery was significantly associated with fecal incontinence at 3 years, with these symptoms occurring more frequently in women who had subsequently delivered by cesarean. This can be attributed to the way in which mode of subsequent delivery following OASIS was determined; women with substantial compromise of anal sphincter function were offered a cesarean delivery [15]. This may also explain why the study showed that having a subsequent vaginal delivery was not a predictor of symptoms in the long term.

In keeping with other studies [15], [19], only 4% of women sustained another sphincter injury in a subsequent vaginal delivery. This information about the low frequency of incontinence symptoms and further tears of the anal sphincter after subsequent vaginal delivery is important for counseling regarding the mode of delivery after OASIS. The findings of the present study differ from those of Nordenstam et al. [4], who found that anal incontinence was significantly more frequent in individuals with a subsequent delivery who had sustained a sphincter tear in the index delivery than in those who had not. However, the numbers with OASIS in that study were very small.

Having a persistent sonographic defect after primary repair of OASIS has been shown to be associated with ongoing incontinence symptoms [20]. In the present study, patients with a persistent defect had no significant change in bowel symptoms; equal numbers of participants had symptoms of fecal urgency, flatus incontinence, and fecal incontinence at early and late follow-ups. By contrast, those without a defect showed a significant improvement in fecal urgency and flatus incontinence. Therefore, if a patient presents with persistent symptoms or new-onset fecal incontinence following OASIS, an endoanal scan should be performed to assess the sphincter. Further management, such as secondary repair or sacral nerve modulation of the sphincters, would then be dependent on multidisciplinary assessment of the severity and degree of burden of symptoms, cosmetic disfigurement of the perineum, presence of an external sphincter defect, and contractility of the residual external sphincter [21].

Assessment of QoL is perhaps a better marker than symptoms alone to determine long-term outcome. In the present study, a validated questionnaire was used to formally assess QoL. Overall, significant improvements with time were recorded in all QoL domains except those of sleep and personal relationships. However, patients with a persistent sphincter defect and those who developed de novo fecal incontinence had no significant improvement in most aspects of QoL. This is confirmatory evidence that persistent anal sphincter defects affect QoL, highlighting the importance of proper primary repair [1].

A limitation of the present study is that, although the patients were referred for training in pelvic muscle exercises if they were symptomatic and had a sphincter defect, the effect of such interventions on long-term symptoms was not quantified. However, a strength of the present study is that it is a large, prospective, long-term study evaluating bowel symptoms following the recommended classification of OASIS [5], [6], [7].

In the present study, the majority of women who were asymptomatic for fecal incontinence at early follow-up remained so after approximately 3 years, and the majority who were initially symptomatic subsequently became asymptomatic. However, 10% developed fecal incontinence for the first time some months or years later, which may be related to fecal urgency and the presence of a persisting sphincter defect. QoL significantly improved unless there was a persisting defect. These findings reinforce the importance of simulation training and one-to-one hands-on teaching about accurate assessment and anal sphincter repair following OASIS [22], [23].
